# Supplementary material for: C-terminal tagging impairs AGO2 function
Source: RNA Biol. 2025 Jul 23;22(1):1–24. doi: 10.1080/15476286.2025.2534028 (PMC12296108; doi:10.1080/15476286.2025.2534028)
Supplement: Supplemental Material [file KRNB_A_2534028_SM0365.docx]

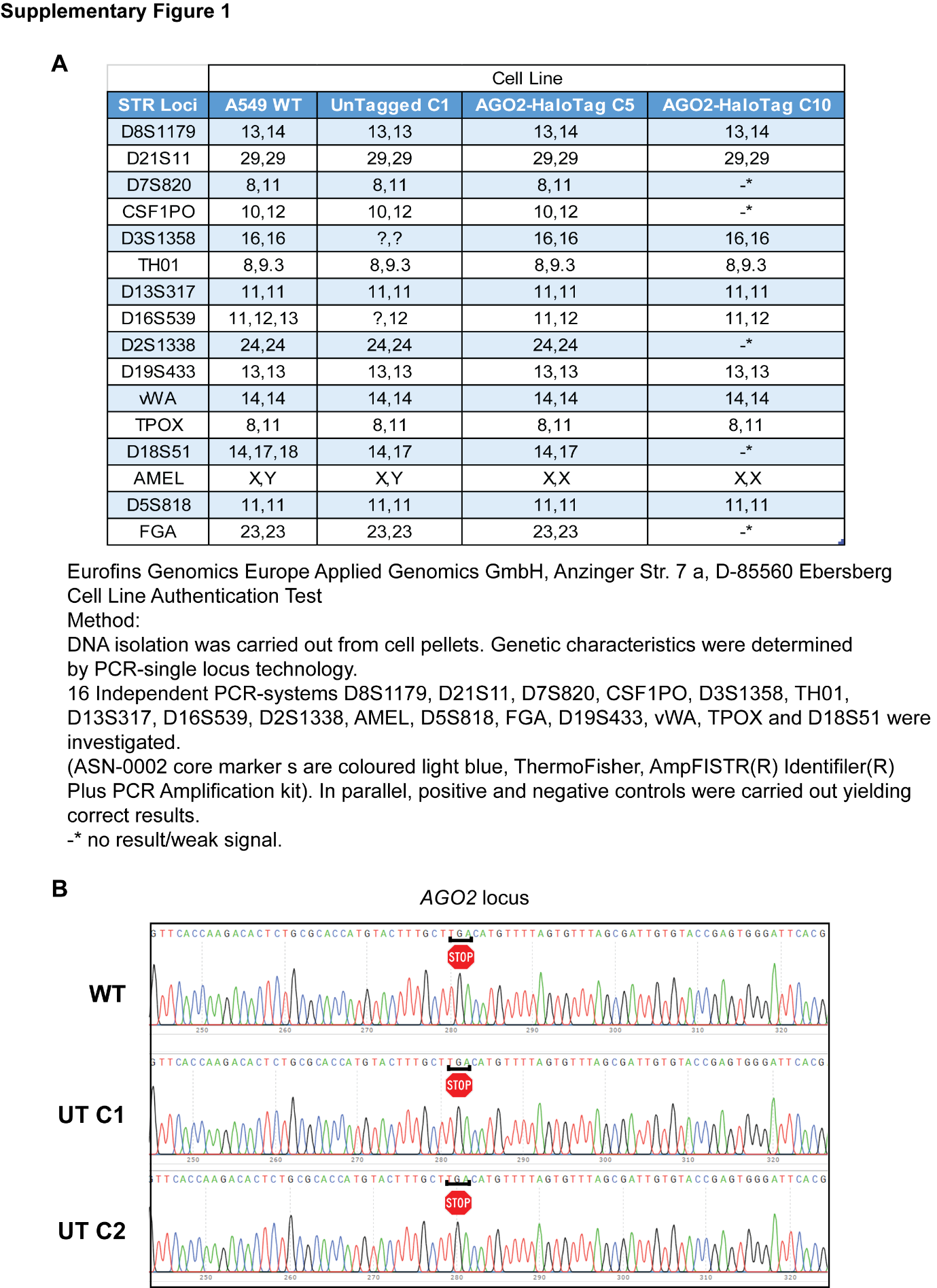


Supplementary Figure 1. STR Profiling for Cell Line Authentication.

(A) Genetic characteristics of indicated cell lines were investigated by STR profiling using 16 independent PCR-systems. Cell Line Authentication Test, Eurofins Genomics Europe Applied Genomics GmbH, Anzinger Str. 7 a, D-85560 Ebersberg

Method: DNA isolation carried out from cell pellet (cell layer). Genetic characteristics were determined by PCR-single-locus-technology. 16 independent PCR-systems D8S1179, D21S11, D7S820, CSF1PO, D3S1358, TH01, D13S317, D16S539, D2S1338, AMEL, D5S818, FGA, D19S433, vWA, TPOX and D18S51 were investigated. In parallel, positive and negative controls were carried out, yielding correct results.

-* no result / weak signal. (B) Genomic DNA sequencing chromatogram traces of the end of the AGO2 locus in UnTagged Clone 1 and Clone 2 cells, showing the DNA is not edited. The same region of AGO2 is shown for WT A549 for comparison.


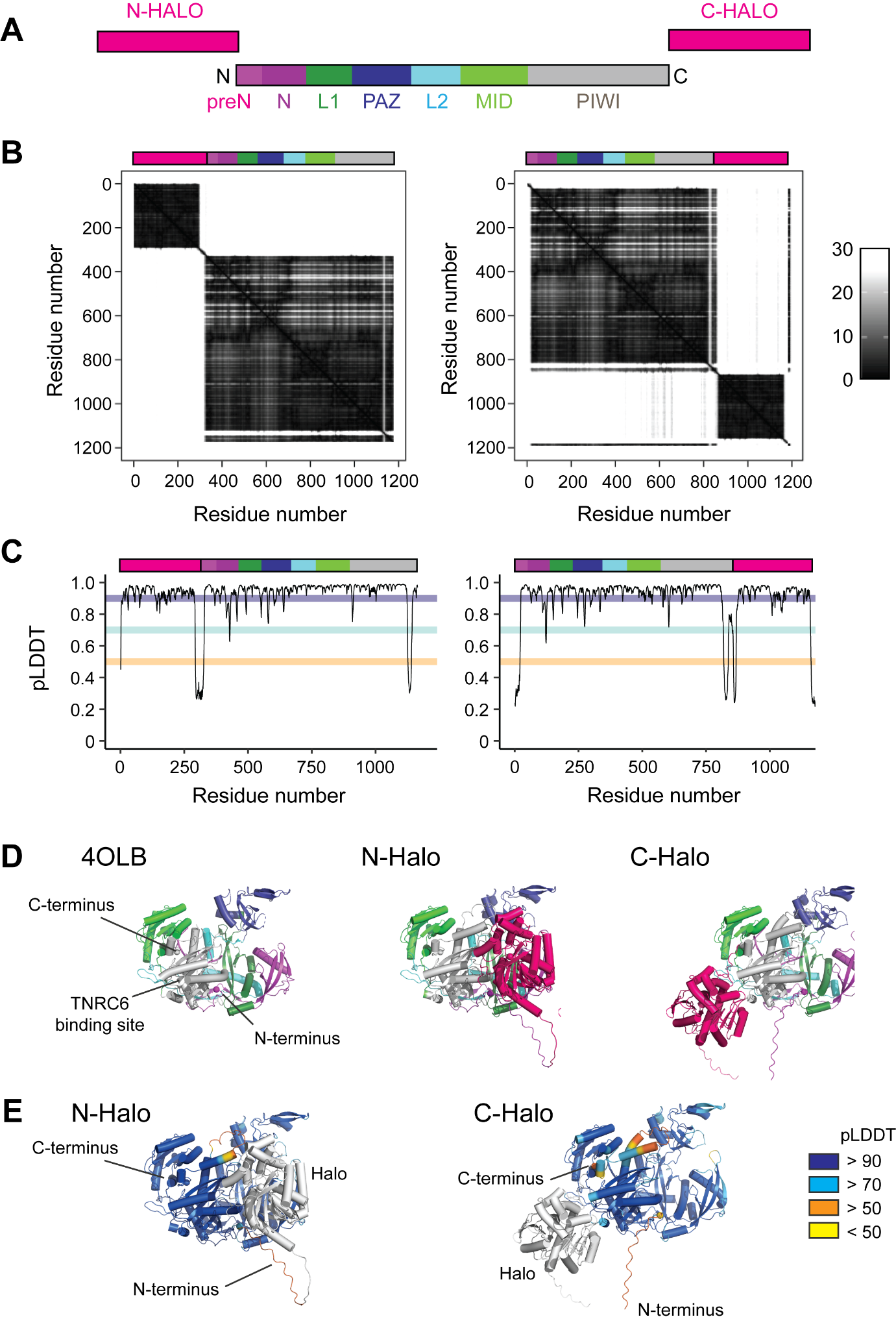


Supplementary Figure 2 AlphaFold3 predictions of N- and C-terminal tagged versions of human AGO2. (A) Schematic composition of human AGO2 showing 7 main domains and motifs and locations of N- or C-terminal fusion of Halo. (B) Predicted alignment error (PAE) and (C) pLDDT plots from AlphaFold3 predictions of the structures of N-terminal (left) or C-terminal (right) fusions of Halo tag to human AGO2. (D) experimental structure of human AGO2 (4OLB) compared to top ranked AlphaFold3 predictions of N-terminal (N-Halo) or C-terminal (C-Halo) tagged constructs. Structures are coloured as in panel (A). N-terminal and C-terminal residues of the experimental structure are shown, as is the location of the TNRC6 binding site. (E) Predicted structure of N-terminal (left) and C-terminal (right) AGO2 constructs with AGO2 coloured by pLDDT value and HaloTag coloured in white.
